# Presidential address: Preparing for permanent test centers and computerized adaptive testing

**DOI:** 10.3352/jeehp.2018.15.1

**Published:** 2018-01-02

**Authors:** Chang Hwi Kim

**Affiliations:** President, Korea Health Personnel Licensing Examination institute, Seoul, Korea; Hallym University, Korea

I am very happy to start the New Year with recent news of progress in the health personnel licensing examinations in Korea.

First, smart device-based testing was used to administer the Korea emergency medical technician licensing examination on December 2, 2017 in 5 cities in Korea, as previously announced [1]. No complaints were received from examinees about taking the examination in this format. To mark answers, examinees touched the corresponding number of each answer item on the screen of a tablet PC, instead of marking an optical mark recognition card. The remaining time was shown on the screen. The examinees said that this format allowed them to concentrate on the test itself. Five items included video files and 26 items presented color graphic files; therefore, practically relevant situations could be tested more easily than on the previous paper and pencil test. From the point of view of examinees’ convenience, smart device-based testing was successful. This result will be a good motivation for extending the scope of smart device-based testing to the licensing examinations for other health professions. Smart device-based testing will be used to administer the Korean medical licensing examination in 2020 [2], which I also anticipate will be successful. Another remarkable event was that 4 Vietnamese delegates from Hue University and Hanoi Open University visited the test sites. They expressed interest regarding the administration methods, item development process, and the activities of the smart device-based testing committee. They proposed a cooperative initiative with our institute to improve the quality of health professionals through not only the adoption of smart device-based testing, but also the introduction of a clinical skills examination. This was an excellent opportunity for our institute to provide assistance to the developers of other countries’ health personnel licensing examinations.

Second, the test construction center of the Korea Health Personnel Licensing Examination Institute was officially opened in Chungju, Korea on June 28, 2017 (Fig. 1). Construction of this center began in March 2017 [1]. The total area (floor space) is 5,057 m^2^ , and it cost 13.8 million dollars. It can provide a working space for 120 persons at the same time, and it will provide test makers with a more comfortable environment in a dedicated place.

In 2018, the institute plans the following new projects: preparing permanent test centers and launching a committee on computerized adaptive testing. Currently, we usually rent test space from high schools to administer paper-and-pencil tests. However, for computer-based testing or computerized adaptive testing to be sustainably implemented, permanent test centers are required in the central cities of each province. The present form of smart device-based testing can be deployed in any location because tablet PCs are easy to handle and move. However, to adopt computerized adaptive testing, the PCs must have a wired internet or intranet connection [3]. Wireless connections to the network was not allowed from tablet PCs for Korea health personnel licensing examinations due to security issues. For this reason, the present form of smart device-based testing involves an offline test, not an online test. After the examination, the answer sheet recorded on the tablet PC is transferred to the server through another form of transfer technology. A special tool for storing and transferring data from tablet PCs must be used. This means that the present form of smart device-based testing is just a replacement of the written test with the merit of presenting video files and having higherquality photos and graphs. If all PCs are connected to the server, it is not necessary to install the same items on each PC and to transfer the answers to the server. Therefore, establishing a browser-based network connection should be the next task to make the administration of computer-based testing more convenient. If we introduce computerized adaptive testing, network connectivity is mandatory. The construction of permanent test centers should be considered for the administration of computerized adaptive testing.

Furthermore, a task force team for computerized adaptive testing was launched in late 2017. As part of the preparation to implement computerized testing, a number of research projects are now under way: first, psychometric considerations, item pool construction, and administrative and financial support for the adoption of computerized adaptive testing; second, test equating of different item sets to implement the test at multiple times. In Korea, where this kind of testing has not been introduced on any high-stakes examinations, such as licensing examinations, we should be aware of the need to persuade not only medical health students and faculty members, but also the general population and government representatives of the merit of computerized adaptive testing as a way to estimate examinees’ ability more efficiently. If we adopt this testing format, examinees will be able to take the tests throughout the year.

I have encouraged staff members of the institute and recipients of support from the institute’s research fund to publish their research output in *Journal of Educational Evaluation for Health Professions*. Last year, 9 of the articles published in the journal described research that had been supported by a research grant from the institute. I hope that more articles supported by grants from the institute will be published in the journal in 2018. I will provide support for the journal to publish continuously as a way of helping medical health educators adopt a variety of innovations related to evaluation. I am also confident that brilliant articles will be published in the journal, making it an essential source of information in the field of educational evaluation.

## Figures and Tables

**Fig. 1.**
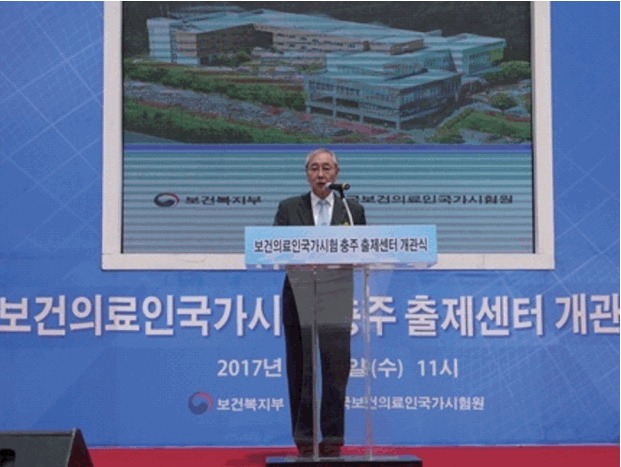
President standing at the opening ceremony of the test construction center of the Korea Health Personnel Licensing Examination Institute in Chungju, Korea on June 28, 2017.
